# Supplementary material for: Understanding variability in petroleum jet fuel life cycle greenhouse gas emissions to inform aviation decarbonization
Source: Nat Commun. 2022 Dec 21;13:7853. doi: 10.1038/s41467-022-35392-1 (PMC9769476; doi:10.1038/s41467-022-35392-1)
Supplement: Supplementary file 1 — Supplementary Information [file 41467_2022_35392_MOESM1_ESM.pdf]

Supplementary Information for

Understanding variability in petroleum jet fuel life cycle Greenhouse gas emissions to inform aviation decarbonization

There are 9 Tables, 9 Figures, and 2 Notes in supplementary information.

Supplementary Table 1 Breakdown of country-level jet fuel well-to-wake CI in 2017. Values are volume-weighted averages in $gCO_2e\ MJ^{-1}$ jet fuel. Note all CI values are first calculated based on domestic jet fuel production and consumption volumes and then adjusted by taking into account the imports (i.e., assigned with the global average well-to-refinery exit emissions). Also note only countries with ≥ 0.2% of the global oil refining and production share are included in the main text.

Country	Extraction	Transportation	Refining	Well-to-Refinery Exit	Distribution	Combustion	Well-to-Wake
Algeria	19.8	0.2	1.0	21.1	<0.1	73.8	94.9
Angola	7.0	0.5	2.1	9.6	0.2	73.8	83.7
Argentina	8.8	0.7	2.1	11.6	0.2	73.8	85.6
Australia	8.7	1.1	3.3	13.1	5.2	73.8	92.2
Austria	10.0	1.0	3.8	14.8	0.2	73.8	88.8
Azerbaijan	5.7	0.3	3.9	9.8	0.2	73.8	83.8
Bahrain	3.3	0.4	4.8	8.5	<0.1	73.8	82.3
Belarus	8.9	1.0	4.2	14.0	0.3	73.8	88.1
Belgium	8.3	1.0	4.3	13.6	1.0	73.8	88.3
Bosnia and Herzegovina	8.4	1.0	4.4	13.7	0.6	73.8	88.1
Brazil	8.5	0.9	3.9	13.4	0.5	73.8	87.7
Bulgaria	9.2	1.0	7.0	17.2	0.1	73.8	91.1
Canada	9.8	1.1	3.9	14.8	2.9	73.8	91.6
Chile	8.6	1.0	4.7	14.3	0.6	73.8	88.8
China	7.9	0.9	6.6	15.4	0.5	73.8	89.7
Colombia	7.7	0.6	1.3	9.6	0.9	73.8	84.2
Cote d`Ivoire	11.6	0.8	3.4	15.8	<0.1	73.8	89.6
Croatia	8.6	0.9	8.3	17.7	0.3	73.8	91.8
Cuba	8.4	1.0	4.4	13.7	0.5	73.8	88.1
Curacao	8.4	1.0	4.4	13.7	0.5	73.8	88.0
Czech Republic	8.0	0.9	4.8	13.7	0.5	73.8	88.1
Denmark	8.1	1.0	4.3	13.4	0.9	73.8	88.1
Ecuador	8.4	0.6	2.6	11.5	0.9	73.8	86.3
Egypt	10.4	0.4	4.5	15.2	0.2	73.8	89.2
Finland	8.5	0.9	4.3	13.8	0.3	73.8	87.8
France	8.9	1.0	4.3	14.2	1.2	73.8	89.2
Germany	8.5	0.9	4.6	14.0	1.2	73.8	89.0
Greece	10.7	0.9	2.7	14.4	0.4	73.8	88.6
Hungary	9.6	0.9	6.8	17.3	<0.1	73.8	91.1
India	9.2	0.9	5.0	15.1	0.3	73.8	89.3
Indonesia	11.1	0.9	2.8	14.8	1.7	73.8	90.2
Iran	14.2	0.5	3.3	18.1	0.2	73.8	92.1
Iraq	10.3	0.7	4.1	15.0	1.4	73.8	90.2

Ireland	7.6	0.9	4.2	12.7	0.6	73.8	87.0
Israel	9.1	0.8	2.5	12.4	0.2	73.8	86.3
Italy	8.3	1.0	4.5	13.8	1.1	73.8	88.7
Japan	6.0	0.8	3.0	9.8	0.3	73.8	83.9
Jordan	4.7	0.6	2.1	7.5	0.3	73.8	81.6
Kazakhstan	8.3	1.7	3.9	13.9	0.8	73.8	88.5
Kuwait	5.3	<0.1	6.0	11.4	<0.1	73.8	85.2
Libya	11.7	0.7	1.8	14.2	0.1	73.8	88.1
Lithuania	8.4	1.0	4.8	14.2	0.9	73.8	88.9
Malaysia	9.3	1.0	2.8	13.1	0.6	73.8	87.5
Mexico	8.0	1.5	3.2	12.6	2.3	73.8	88.8
Netherlands	8.4	1.0	4.6	13.9	1.1	73.8	88.8
New Zealand	6.5	1.8	4.0	12.3	0.8	73.8	86.9
Nigeria	9.2	0.7	3.5	13.4	1.1	73.8	88.3
Norway	4.1	0.3	2.0	6.4	0.9	73.8	81.0
Oman	9.6	0.7	7.5	17.8	0.3	73.8	91.9
Pakistan	6.4	0.7	3.2	10.3	0.9	73.8	85.0
Peru	9.1	0.7	2.6	12.4	0.7	73.8	86.8
Philippines	7.0	0.9	3.2	11.1	1.2	73.8	86.1
Poland	8.8	1.0	8.0	17.8	0.2	73.8	91.7
Portugal	8.6	0.9	2.7	12.2	1.1	73.8	87.1
Qatar	6.1	0.4	1.2	7.7	<0.1	73.8	81.5
Romania	7.6	0.6	3.9	12.1	0.3	73.8	86.2
Russian Federation	8.7	0.6	3.2	12.5	0.1	73.8	86.5
Saudi Arabia	2.9	0.4	4.5	7.9	0.1	73.8	81.7
Serbia	10.7	1.0	6.6	18.2	0.2	73.8	92.2
Singapore	6.8	0.9	3.3	11.0	0.5	73.8	85.2
Slovakia	8.5	0.9	12.5	21.9	<0.1	73.8	95.7
South Africa	6.8	0.8	3.9	11.5	0.5	73.8	85.8
South Korea	6.7	1.0	3.5	11.1	0.1	73.8	85.0
Spain	9.2	1.0	5.5	15.7	1.0	73.8	90.6
Sudan	10.8	0.4	2.3	13.5	1.0	73.8	88.4
Sweden	8.5	1.0	4.1	13.6	1.6	73.8	89.0
Switzerland	8.4	1.0	4.4	13.8	1.0	73.8	88.6
Taiwan	6.6	0.9	2.9	10.4	0.1	73.8	84.3
Thailand	6.4	0.7	3.3	10.4	0.3	73.8	84.5
Trinidad and Tobago	9.4	1.2	0.9	11.5	<0.1	73.8	85.3
Turkey	13.0	0.9	6.7	20.6	0.4	73.8	94.8
Turkmenistan	14.5	<0.1	2.6	17.1	0.6	73.8	91.5
Ukraine	10.3	0.5	0.9	11.7	0.2	73.8	85.7

United Arab Emirates	7.3	0.5	2.0	9.8	0.3	73.8	83.9
United Kingdom	8.2	0.9	3.9	13.1	1.3	73.8	88.2
United States	9.7	1.5	5.2	16.4	0.6	73.8	90.8
Uruguay	10.3	1.1	1.0	12.4	<0.1	73.8	86.2
Uzbekistan	27.6	<0.1	1.1	28.7	0.3	73.8	102.8
Venezuela	18.3	2.0	0.9	21.2	<0.1	73.8	95.0
Vietnam	7.8	0.7	4.2	12.7	0.7	73.8	87.2
**Global Average**	**8.6**	**1.0**	**4.5**	**14.1**	**0.8**	**73.8**	**88.7**

Supplementary Table 2 A summary of refinery-level jet fuel refining. Note only 397 refineries that produce jet fuel are included. Jet fuel refining CI are not adjusted by import or export. The numbers of refineries that produce CDU jet, KHT jet, DHT jet, and DHCU jet are 189, 128, 162, 111, respectively. Refinery crude assay jet vol% is the volume fraction of jet fuel contained in the crude blend of each refinery (converted based on PRELIM pre-determined temperature cuts). Refinery jet vol% measures the actual volumetric production of jet fuel. See Extended Data Fig. 6 for a correlation matrix.

		Global	North America	Latin America	The Middle East	Asia & Oceania	Europe & Russia	Africa
Refinery Nelson Complexity	*5th percentile*	3.69	6.68	3.98	2.77	4.91	4.13	1.48
	95th percentile	13.7	16.0	9.60	9.47	13.0	11.4	8.84
Refinery Crude mix API (°)	*5th percentile*	25.2	24.2	22.0	28.3	29.2	31.4	30.9
	95th percentile	41.4	42.2	34.4	45.4	40.1	39.8	45.7
	v.w.a.	33.5	32.9	26.9	34.9	33.8	34.9	36.9
Refinery Crude mix Sulfur Content (wt.%)	*5th percentile*	0.12	0.21	0.26	0.57	0.08	0.24	0.08
	95th percentile	2.09	2.32	1.75	2.57	1.93	1.87	1.28
	v.w.a.	1.14	1.19	0.80	1.56	1.20	0.92	0.57
Refinery Crude mix Jet Content (vol.%)	*5th percentile*	16.2	16.1	13.7	16.9	16.1	19.6	19.1
	95th percentile	25.9	26.0	25.4	25.4	25.7	24.8	27.8
	v.w.a.	21.1	21.0	19.5	20.8	21.0	21.7	23.2
WM Refinery Jet Yield (vol.%)	*5th percentile*	1.90	2.78	0.98	3.68	1.86	1.24	2.92
	95th percentile	20.2	17.3	14.4	23.3	20.2	19.8	26.5
	v.w.a.	9.70	9.19	6.87	12.9	10.4	8.25	12.9
Jet Refining CI (g CO_2e MJ^{-1} jet)	*5th percentile*	0.93	1.11	0.77	0.83	0.99	0.92	0.75
	95th percentile	9.37	9.35	7.10	8.16	9.75	9.41	5.63
	v.w.a.	4.40	5.14	2.66	3.94	4.20	4.68	3.15
CDU Jet Refining CI (g CO_2e MJ^{-1} jet)*	*5th percentile*	0.74	0.79	0.66	0.72	0.83	0.73	0.65
	95th percentile	1.20	1.07	0.99	1.04	1.22	0.97	0.99
	v.w.a.	0.92	1.01	0.86	0.80	0.98	0.83	0.89
KHT Jet Refining CI (g CO_2e MJ^{-1} jet)*	*5th percentile*	0.73	0.88	0.67	0.70	0.83	0.69	0.67
	95th percentile	5.13	5.15	4.88	4.15	5.19	4.13	3.52
	v.w.a.	4.56	4.81	4.86	3.98	4.70	3.89	3.20
DHT Jet Refining CI (g CO_2e MJ^{-1} jet)*	*5th percentile*	3.39	4.60	3.85	2.90	4.01	3.16	3.45
	95th percentile	9.43	10.3	8.72	6.59	9.78	8.07	6.28
	v.w.a.	6.29	7.72	7.15	4.75	7.38	5.55	5.21
DHCU Jet Refining CI (g CO_2e MJ^{-1} jet)*	*5th percentile*	6.17	5.64	10.4	6.52	8.09	6.57	9.51
	95th percentile	13.3	12.0	11.6	12.3	13.7	12.3	9.97
	v.w.a.	10.5	9.66	10.7	9.79	11.5	10.4	9.64

*Only includes refineries that produce this type of jet fuel.

Supplementary Table 3 Volumetric composition of jet fuel by region.

Configuration	CDU Jet (%)	KHT Jet (%)	DHT Jet (%)	DHCU Jet (%)
Global	37.2	25.6	21.2	16.0
North America	19.8	43.5	19.7	17.0
Latin America	61.0	21.9	15.2	1.9
The Middle East	43.7	7.1	27.6	21.6
Asia & Oceania	47.6	25.5	11.9	15.0
Europe & Russia	26.6	19.5	37.1	16.8
Africa	49.4	20.2	20.7	9.7

Supplementary Table 4 Volumetric composition of jet fuel by country. Dash line indicates no data available.

Country	CDU Jet (%)	KHT Jet (%)	DHT Jet (%)	DHCU Jet (%)
Algeria	100	0	0	0
Angola	68.1	31.9	0	0
Argentina	89.1	0	0	10.9
Australia	100	0	0	0
Austria	0	100	0	0
Azerbaijan	0	100	0	0
Bahrain	42.8	0	30.7	26.5
Belarus	0	51.6	48.4	0
Belgium	60.4	39.6	0	0
Bosnia and Herzegovina	-	-	-	-
Brazil	16.4	61.5	22.2	0
Bulgaria	0	0	100	0
Canada	26.7	0	45.5	27.7
Chile	38	0	55	7
China	17.7	29.6	25.8	27
Colombia	100	0	0	0
Cote d`Ivoire	69.6	0	0	30.4
Croatia	0	0	0	100
Cuba	-	-	-	-
Curacao	-	-	-	-
Czech Republic	0	0	100	0
Denmark	0	38.5	61.5	0
Ecuador	100	0	0	0
Egypt	28.8	28.7	9.9	32.5
Finland	47	0	28.4	24.6
France	5.8	61.4	32.7	0
Germany	12.5	29.3	50.5	7.7
Greece	75.1	0	9.3	15.7
Hungary	0	0	100	0
India	42.5	20.2	15.1	22.1
Indonesia	87.3	0	12.7	0
Iran	36.1	19.5	38.1	6.4
Iraq	0	100	0	0
Ireland	0	0	100	0
Israel	20.7	29.4	49.9	0
Italy	12.8	52.4	31.6	3.2
Japan	50.4	44.2	4.1	1.4
Jordan	82	0	0	18
Kazakhstan	5.1	0	94.9	0
Kuwait	0	0	70.5	29.5
Libya	62.7	37.3	0	0
Lithuania	0	0	100	0
Malaysia	92.4	0	2.8	4.8
Mexico	79.8	0	20.2	0
Netherlands	0	41.6	14.5	43.9
New Zealand	55.7	0	0	44.3
Nigeria	28.5	71.5	0	0

Norway	81.3	0	18.8	0
Oman	13.7	28.9	0.7	56.7
Pakistan	76.5	0	0	23.5
Peru	100	0	0	0
Philippines	89.6	0	10.4	0
Poland	0	0	72.3	27.7
Portugal	62.4	0	37.6	0
Qatar	90.2	0	9.8	0
Romania	0	100	0	0
Russian Federation	43.6	8.3	40	8.1
Saudi Arabia	42	0	24.6	33.3
Serbia	0	0	100	0
Singapore	76.1	0	5.3	18.6
Slovakia	0	0	0	100
South Africa	30.9	17.2	51.9	0
South Korea	49.3	40.5	4.4	5.7
Spain	15.9	6.2	54.6	23.2
Sudan	100	0	0	0
Sweden	91.4	0	0	8.6
Switzerland	0	0	100	0
Taiwan	76.3	0	12.4	11.3
Thailand	57.6	14.4	5.2	22.7
Trinidad and Tobago	100	0	0	0
Turkey	0	0	61.5	38.5
Turkmenistan	48.3	0	51.7	0
Ukraine	100	0	0	0
United Arab Emirates	92.2	0	2.8	5.1
United Kingdom	69.9	1.2	20.9	8
United States	19.7	44.8	18.9	16.6
Uruguay	100	0	0	0
Uzbekistan	100	0	0	0
Venezuela	100	0	0	0
Vietnam	0	100	0	0
**Global Average**	37.2	25.6	21.2	16

Supplementary Table 5 Percentage of refineries that produce CDU jet only, KHT and/or DHT jet only, DHCU Jet only, and mixed jet fuel streams. Numbers in the parentheses after configuration names are the total numbers of refineries that produce jet fuel in 2017.

Configuration	CDU Jet only (%)	KHT and/or DHT Jet only (%)	DHCU Jet only (%)	Mix of streams (%)
Hydroskimming (39)	46	38	0	16
Medium Conversion (179)	33	31	4	32
Deep Conversion Coking (166)	22	31	6	41
Deep Conversion Hydrocracking (14)	14	50	14	22
**Region**				
North America (91)	12	35	7	46
Latin America (25)	56	16	4	24
The Middle East (29)	31	17	7	45
Asia and Oceania (136)	39	22	3	36
Europe and Russia (97)	20	54	6	20
Africa (20)	45	30	0	25
Global (398)	29	32	5	34

Supplementary Table 6 Country-wise indirect emissions associated with natural gas. Indirect emissions include emissions emitted during extraction, processing, and transportation from well to refinery entrance.

Country	Indirect Emissions Intensity ($gCO_2e\ MJ^{-1}$)	Emissions Intensity Range ($gCO_2e\ MJ^{-1}$)	Reference
Australia	8.4	7.8-9	[1]
Brazil	20	-	[2]
Canada	6.4	6.1-16.8	[3,4]
China	21.7	6.2-43.3	[1]
Germany	11	10.0-12	[3,5]
India	15.7	-	[2]
Iran	13.2	-	[2]
Italy	15.7	-	[2]
Japan	11.2	-	[6]
Nigeria	6.1	-	[7]
Norway	6.1	-	[3]
Oman	27.9	26-29.7	[1]
Qatar	4	-	[3]
Russian Federation	9.9	7.3-11.6	[1]
Saudi Arabia	6.2	-	[2]
South Korea	14.4	-	[8]
Spain	15.7	-	[2]
Turkmenistan	7.6	6.7-8.5	[1]
United Kingdom	5	-	[9]
United States	15	7.2-27.2	[1,3,4]
Uzbekistan	16.5	-	[1]
Others	10.8	6-15	[10]

Supplementary Table 7 Electricity emissions factor for Canada in 2017.

Category	*g CO_2eq/kWh*
CO_2	142
CH_4	0.1
N_2O	0.6
Total	142.7
Correction for transmission and distribution losses	16.4
trade induced emissions	4.1
Average after correction	**163.2**

Supplementary Table 8 Summary of key parameter ranges used for sensitivity analysis of jet fuel refining CI. The lower and upper bounds of SMR PSA hydrogen production emissions are based on the Greenhouse Gases, Regulated Emissions, and Energy Use in Technologies Model (GREET 2013 and 2019) by Argonne National Laboratory. Allocation methods tested in this study include process-level hydrogen-, mass-, and price-based as well as refinery-level energy, hydrogen-, mass-, and price-based allocation. The marginal emissions associated with jet fuel refining are not calculated as a comparator because the refinery linear programming (LP) models are not available but are similar to those of the process-unit energy allocation results from Moretti et al. (2017)[11].

**Parameter**	**Unit**	**Baseline Value**	**Lower Bound**	**Upper Bound**	**Reference**
Indirect emissions factor for natural gas	g CO_2eq MJ^{-1} natural gas	See Supplementary Table 6			
Process energy required by key process units	-	PRELIM default	PRELIM lower bound	PRELIM upper bound	[10]
SMR PSA H_2 emissions	kg CO_2e/kg H_2	10.4	9	11.9	[12,13]
Carbon capture efficiency	%	85	80	90	[14–16]
Allocation Method	-	Process-level energy based	-	-	-

Supplementary Table 9 Sustainable aviation fuel (SAF) required for international aviation carbon neural growth out to 2050 under different petroleum jet fuel supply chain decarbonization scenarios. MSW: municipal solid wastes. NBC: non-biogenic carbon content. Technology improvements and ATM/infrastructure improvements are included for all scenarios. The well-to-wake petroleum baseline of 88.7 gCO_2e MJ^{-1} is adopted for calculations. The energy contents of FT, HEFA, SIP, ATJ, and ETJ SAFs are assumed to be 44.2, 43.4, 43.2, 43.2, and 43.2 MJ/kg, respectively[17–19].

**Fuel Conversion Process**	**Feedstock**	**Default core LCA value approved by ICAO (gCO_2e MJ^{-1})**
Fischer-Tropsch (FT)	Agricultural residues	7.7
	Forestry residues	8.3
	MSW (0% NBC)	5.2
	MSW (40% NBC)	73.4
	Short-rotation woody crops	12.2
	Herbaceous energy crops	10.4
Hydroprocessed esters and fatty acids (HEFA)	Tallow	22.5
	Used cooking oil	13.9
	Palm fatty acid distillate	20.7
	Corn oil	17.2
	Soybean oil	40.4
	Rapeseed oil	47.4
	Camelina	42
	Palm oil (close pond)	37.4
	Palm oil (open pond)	60
	Brassica carinata	34.4
Synthesized iso-paraffins (SIP)	Sugarcane	32.8
	Sugarbeet	32.4
Iso-butanol alcohol-to-jet (Iso-BuOH ATJ)	Sugarcane	24
	Agricultural residues	29.3
	Forestry residues	23.8
	Corn grain	55.8
	Herbaceous energy crops	43.4
	Molasses	27
Ethanol-to-jet (ETJ)	Sugarcane	24.1
	Corn grain	65.7

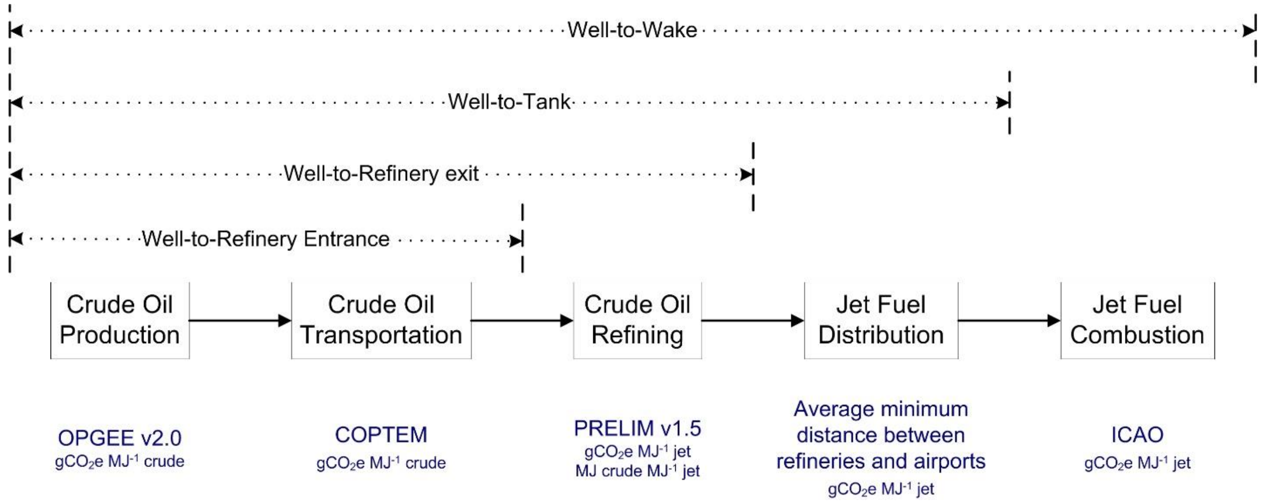

Supplementary Fig. 1 The LCA system boundary of jet fuel. The upper portion shows life cycle stages, while the lower part states models and methods used to calculate life cycle GHG emissions of jet fuel.

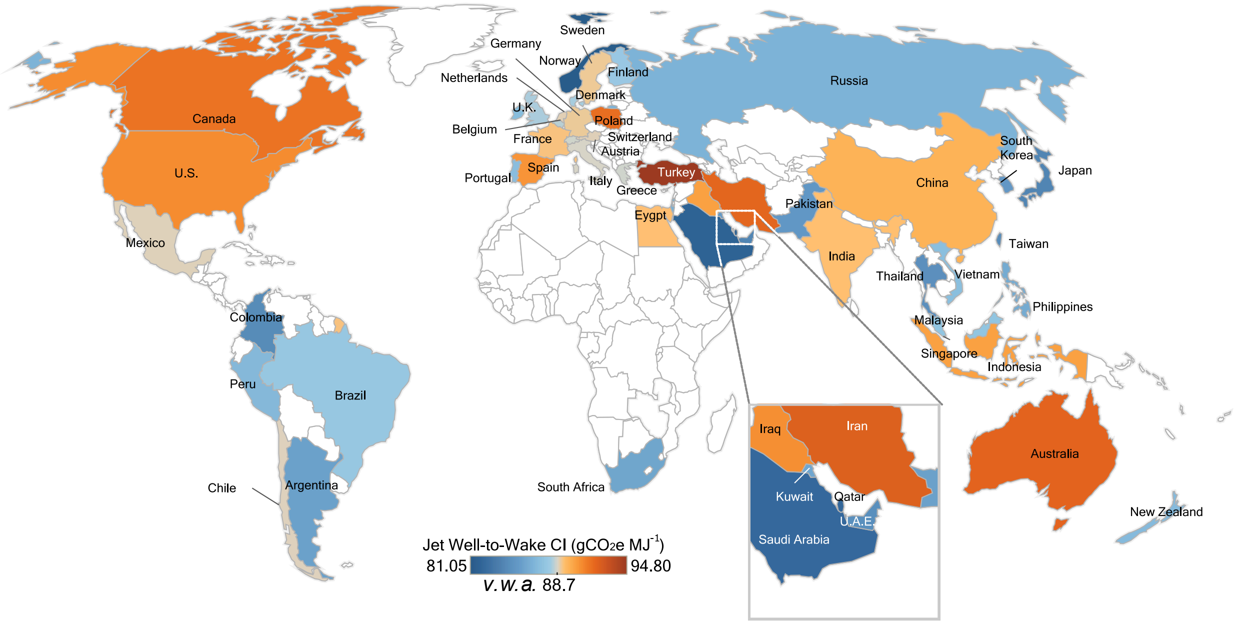

Supplementary Fig. 2 Jet fuel volume-weighted average well-to-wake CI in 2017 by country. The global volume-weighted average well-to-wake CI is 88.7 gCO_2e MJ^{-1}. Each country's volume-weighted average CI is estimated by considering the volume weighting of domestic production and imported jet fuel CI (this is also done in Fig. 2b, see Methods for details). Only countries with ≥ 0.2% of the global oil refining and production share are assigned a color corresponding to a volume-weighted average CI intensity. Countries with white-colored backgrounds are not included in this study because their refining volume is lower than the 0.2% cut-off threshold or no data is available.

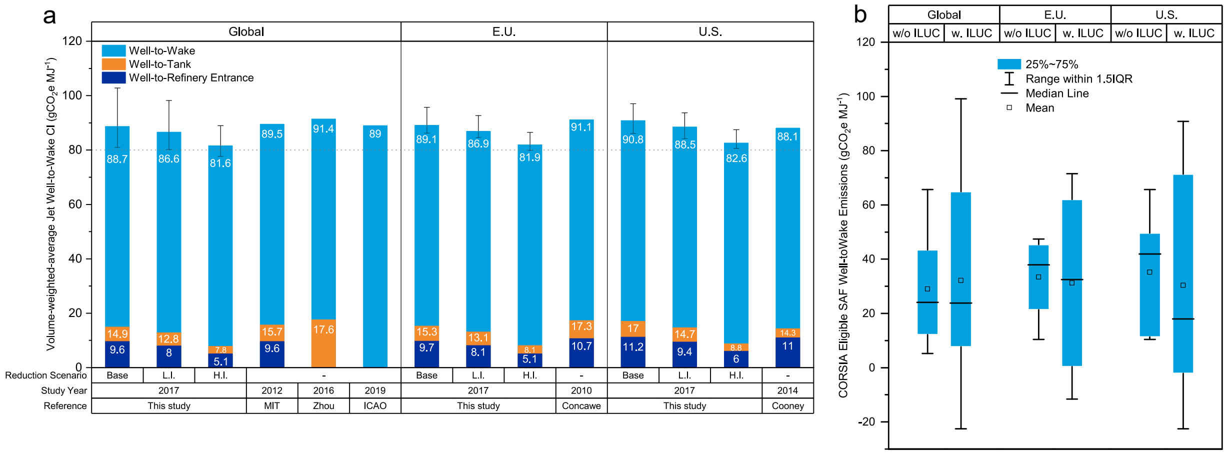

Supplementary Fig. 3 Comparison between results obtained from this study and a) petroleum jet fuel well-to-wake CI values reported in the literature and b) sustainable aviation fuel (SAF) reported by ICAO. In subplot a), study year refers to the period for which data are collected; reduction scenarios L.I. and H.I. are fuel supply chain low and high technology improvements scenarios, respectively (see Methods); error bars are 5 and 95 percentiles; the numbers highlighted are cumulative life cycle emissions, for example, the first stacked column has a well-to-refinery entrance CI of 9.6 $gCO_2e\ MJ^{-1}$ jet fuel, a well-to-tank CI of 14.9 $gCO_2e\ MJ^{-1}$ jet fuel (indicating refining and distribution in total has 5.3 $gCO_2e\ MJ^{-1}$ jet fuel), and a well-to-wake CI of 88.7 $gCO_2e\ MJ^{-1}$ jet fuel (indicating a combustion CI of 73.8 $gCO_2e\ MJ^{-1}$ jet fuel). Numbers are cited from MIT[20], Zhou et al.[21], ICAO[22], Concawe[11,23], and Cooney et al[24]. In subplot b), ILUC stands for induced land use change. Numbers are taken from ICAO[25].

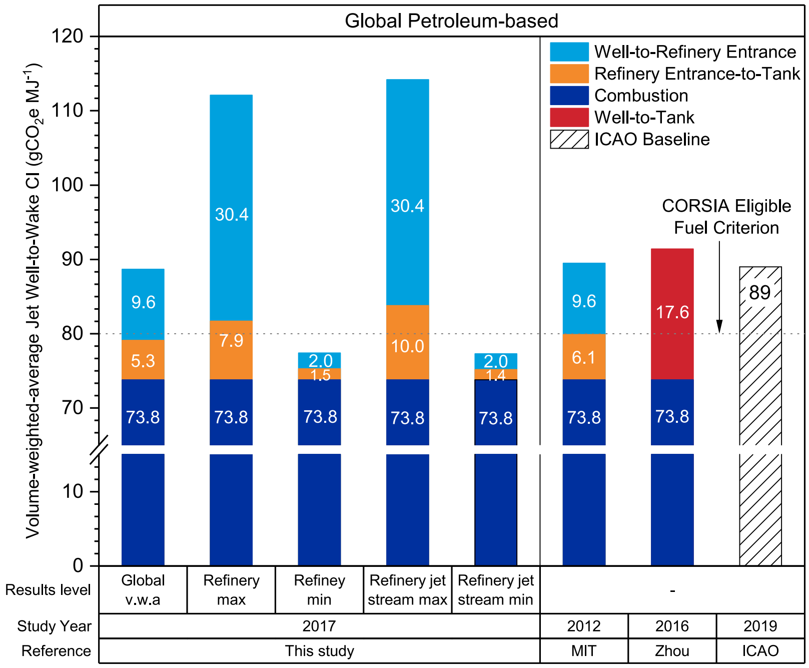

Supplementary Fig. 4 Comparison between results obtained from this study and petroleum jet fuel well-to-wake CI values reported in the literature. Study year refers to the period for which data are collected. Global v.w.a. stands for the global volume-weighted average CI (88.7 $gCO_2e\ MJ^{-1}$). Refinery max and min are the highest and lowest well-to-wake CI of jet fuel produced from all refineries. Refinery jet stream max and min are the highest and lowest well-to-wake CI of all four types of jet fuel produced from the atmospheric unit (CDU jet), kerosene hydrotreater (KHT jet), diesel hydrotreater (DHT jet), and the distillate hydrocracking unit (DHCU jet). Refinery max and min, and refinery jet stream max and min include the production and transportation CI of a refinery's crude feedstock, jet fuel refining CI, and an average jet fuel distribution CI within the country where this refinery is located, and combustion CI. Note import and export are not considered for refinery level results. See references for studies conducted by MIT[20] and Zhou et al.[21] and note combustion emissions are set as 73.8 $gCO_2e\ MJ^{-1}$ for these two studies.

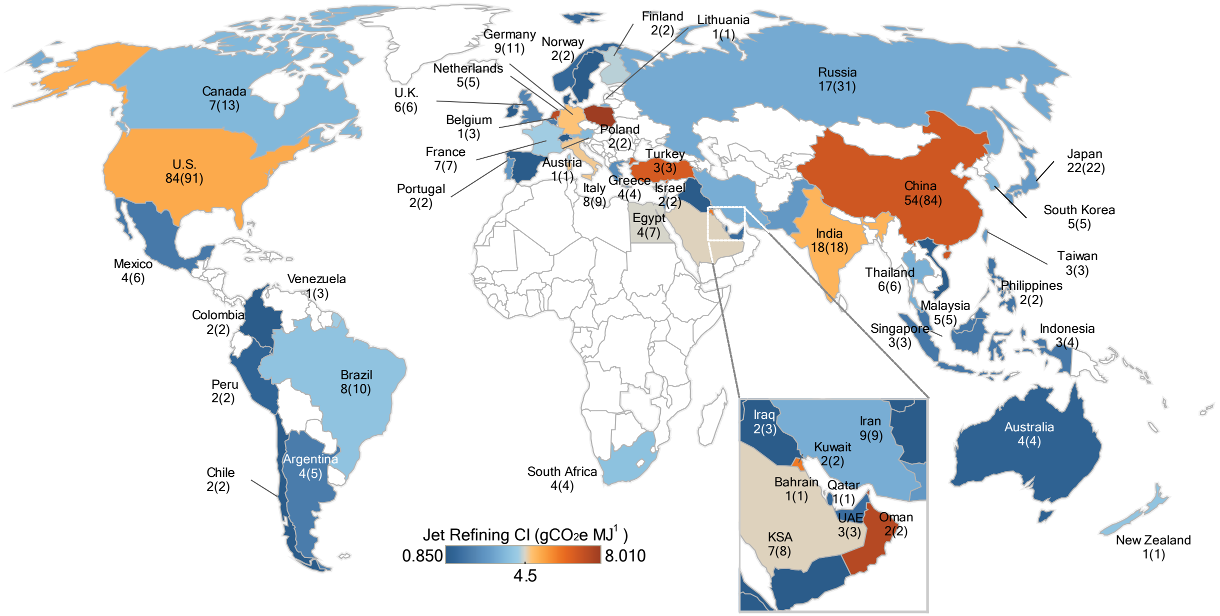

Supplementary Fig. 5 Country-level jet fuel refining CI. The numbers below country names are the number of refineries that produce jet fuel, followed by the total number of refineries (in parentheses). The global volume-weighted average is 4.5 $gCO_2e\ MJ^{-1}$. For clarity, only countries with ≥ 0.2% of global oil refining and production share are mapped. Countries with white-colored backgrounds are either not included in this study, or refining volume is < 0.2% cut-off threshold.

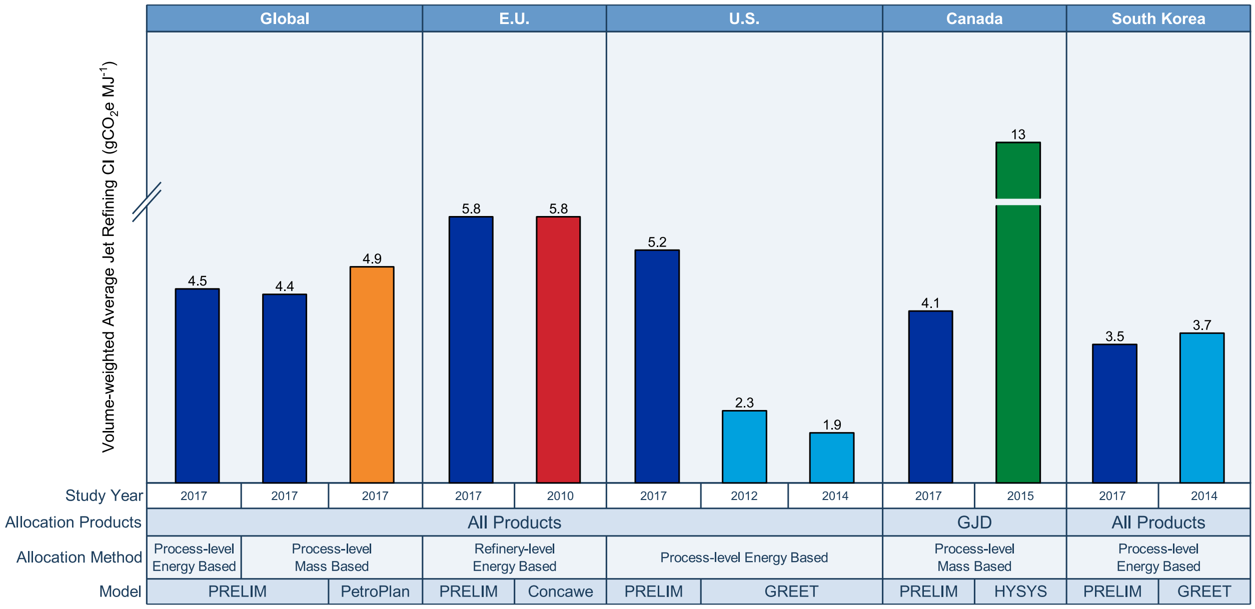

Supplementary Fig. 6 Comparison between results obtained from PRELIM and jet fuel refining CI values reported in the literature. All dark blue bars are results obtained from PRELIM, including the first one being this study's global volume-weighted average, and the remaining five being PRELIM results with adjusted allocation settings to match the corresponding literature studies. Study year refers to the period for which data are collected. Allocation products refer to the products considered for energy and emissions allocation. GJD means only gasoline, jet fuel, and diesel are included for allocation. The allocation method can be either process unit-level or refinery-level, differentiated by allocating energy and emissions to intermediate streams (and then aggregated for final products) or directly to final products. Energy- and mass-based allocation are used to apportion the energy and emissions burden based on energy content and mass of the streams/products, respectively. PetroPlan results are available from the Wood Mackenzie datasets[26,27]; Concawe results are based on Moretti et al.[11]; the Greenhouse Gases, Regulated Emissions, and Energy Use in Technologies Model (GREET) results are from studies conducted by the Argonne National Laboratory and Jang and Song[28–30]; HYSYS results are from Nimana et al. [31].

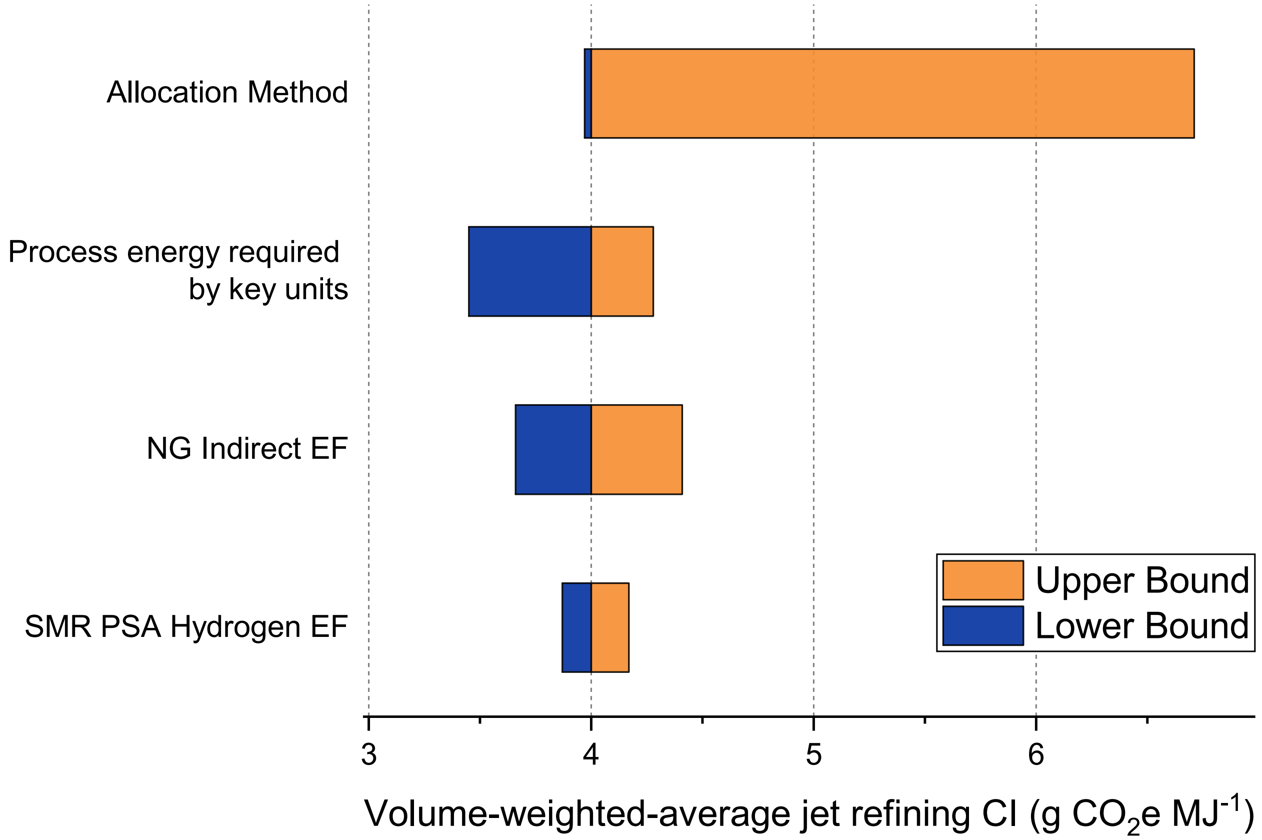

Supplementary Fig. 7 Sensitivity analysis tornado plot of selected parameters. The upper and lower bounds are listed in Supplementary Table 8. Also, see Extended Data Fig. 3 for details about the allocation method. Note the base case refining CI is 4.02 gCO_2e MJ^{-1} because only 100 refineries are included here. Results from the other two process-unit-level allocation methods (i.e., 3.99 gCO_2e MJ^{-1} from the hydrogen-based and mass-based allocation methods as shown by the lower bound) are close to the base case CI. Contrastingly, the refinery-level allocation methods have higher refining CI ranging from 4.73 (the price-based allocation) to 6.73 (the hydrogen-based allocation as shown by the upper bound) gCO_2e MJ^{-1}. This is because jet fuel, in general, is produced through fewer units than other major refining products (e.g., gasoline and diesel). Therefore, its refining CI tends to be overestimated by the refinery-level allocation methods. The low- and high-technology improvement scenarios can reduce jet fuel refining CI to 3.67 and 1.76 gCO_2e MJ^{-1}, respectively. Although carbon capture efficiency is not included here, a decrease of capture efficiency from 85% (base case) to 80% (Supplementary Table 8) can increase the jet fuel refining CI to 3.70 and 1.92 gCO_2e MJ^{-1} under the low- and high-technology improvement reduction scenarios, respectively. Similarly, an increase in capture efficiency from 85% (base case) to 90% can reduce the jet fuel refining CI to 3.65 and 1.60 gCO_2e MJ^{-1} under the low- and high-technology improvement reduction scenarios.

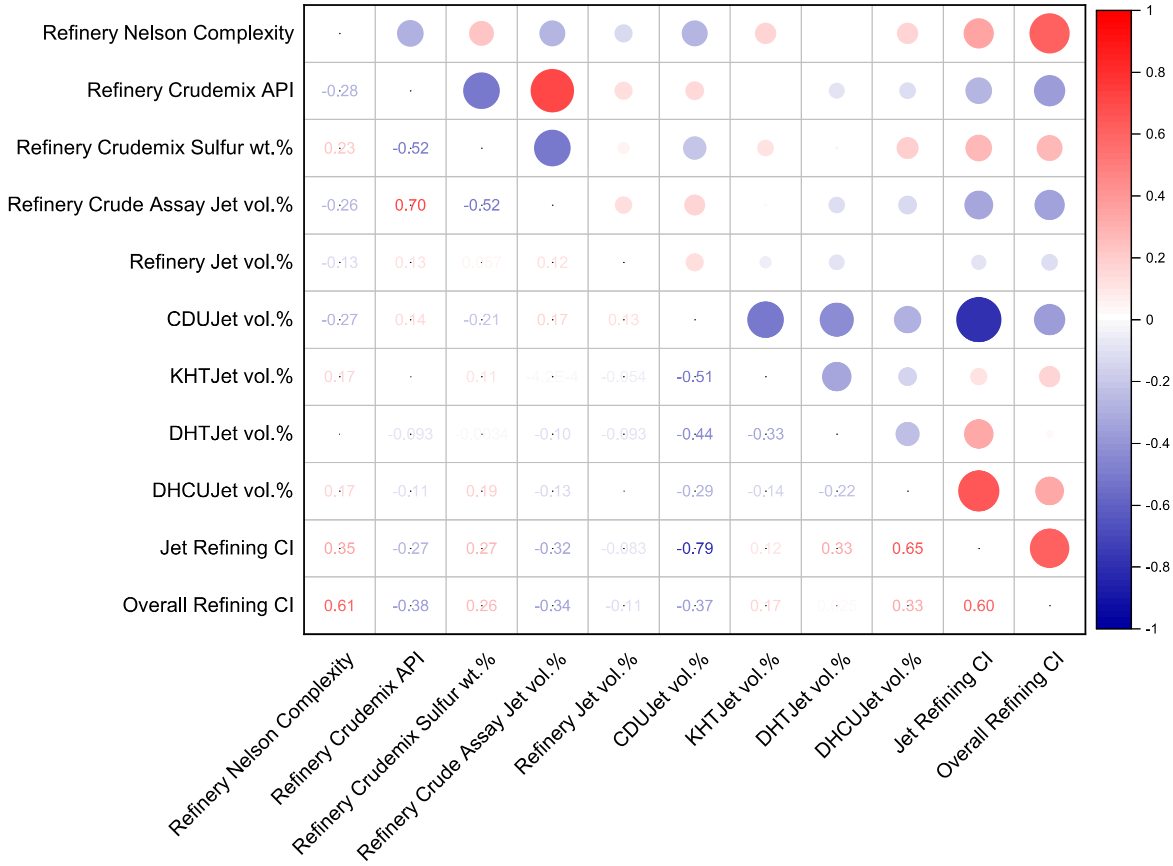

Supplementary Fig. 8 Correlation matrix plot for jet fuel refining CI, refinery Nelson complexity, quality of crude refined, and volume shares of different types of the jet fuel blend. Jet fuel refining CI results are obtained from each refinery. Refinery crude assay jet vol% is the volume fraction of jet fuel contained in the crude blend of each refinery (converted based on PRELIM pre-determined temperature cuts). Refinery jet vol% measures the actual volumetric production of jet fuel. Overall refining CI is the refinery-level emissions intensity in kgCO_2e bbl^{-1} crude oil. The numbers in the bottom left half are Pearson correlation coefficients. It can be noted that jet fuel refining CI is highly positively correlated with DHCUjet vol.% and highly negatively correlated with CDUjet vol.%.

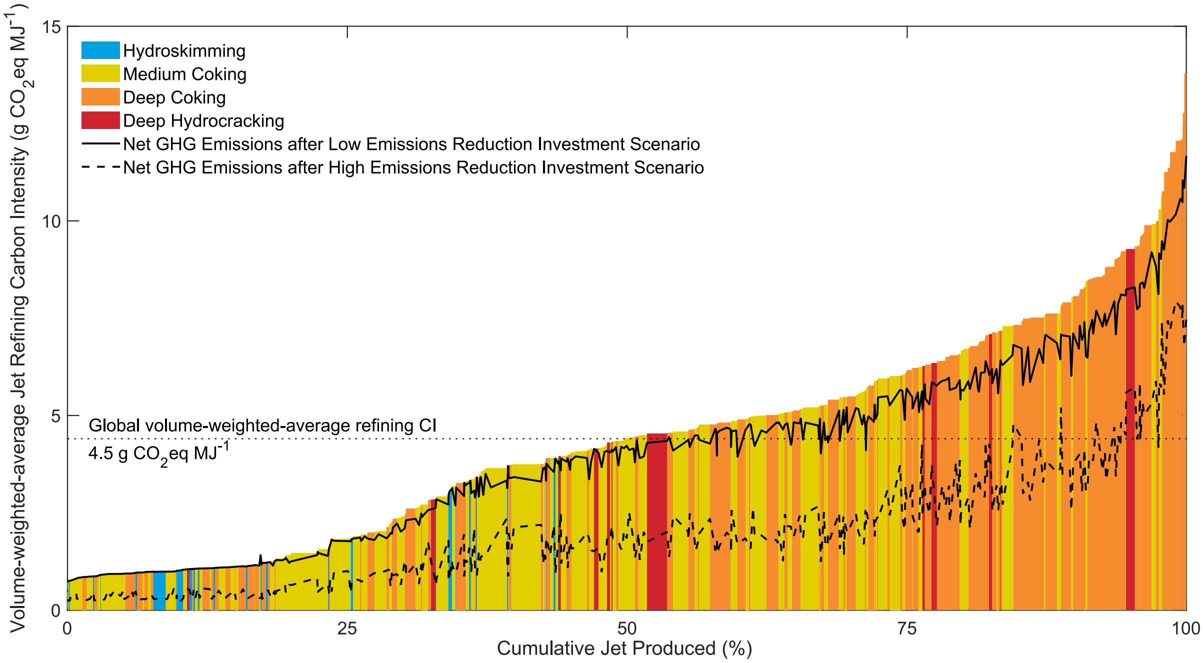

Supplementary Fig. 9 Global jet fuel refining CI by refinery in 2017. Each vertical bar represents a refinery that produces jet fuel. Bar width represents the daily volume of jet fuel produced. Bars are sorted by increasing refining CI. The global volume-weighted average refining CI can be reduced to 4 and 2 gCO_2e MJ^{-1} under low- and high-technology improvement scenarios. More details about emissions reduction scenarios can be found in Methods.

Supplementary Note 1

Hydroskimming is the simplest refinery configuration in PRELIM. It includes atmospheric distillation and final products hydrotreating.

Medium conversion is hydroskimming plus vacuum distillation and fluid catalytic cracking (FCC) and/or distillate hydrocracker (DHCU). There are three configurations in this category: medium conversion FCC, medium conversion DHCU, and medium conversion FCC+DHCU.

Deep conversion coking includes medium conversion units plus a delayed coking unit. There are three configurations in this category: deep coking FCC, deep coking DHCU, and deep coking FCC+DHCU.

Deep conversion hydrocracking includes medium conversion units plus a residue hydrocracking unit. There are three configurations in this category: deep hydrocracking FCC, deep hydrocracking DHCU, and deep hydrocracking FCC+DHCU.

More details can be found in the literature[10,32].

Supplementary Note 2

The aviation industry’s four-pillar strategy for climate change includes technology improvements, operations and infrastructure improvements, carbon offsets and sustainable aviation fuel (SAF). Technology improvements refers to the development of new aircraft and engines (e.g., electric- and hydrogen-powered engines) that are superior in propulsion, aerodynamics, weight reduction, and urban air mobility. New technologies on average can save 15%-20% of current fuel use. Operations improvements refer to the weight saving, single-engine taxing, idle reverse thrust, continuous descents into airport, traffic flow management and other ATC procedures that can reduce airborne holding time. Infrastructure improvements include navigation improvements, better use of air space, better streamlining of routes, flight time reduction, and airport layout optimization to reduce aircraft holding time. Carbon offsets the global market-based measure ICAO has adopted, namely Carbon Offsetting and Reduction Scheme for International Aviation (CORSIA). SAF can be produced from alternative sources (e.g., biomass) and directly used as drop-in fuels without any engine or system modifications. SAF have already been used in more than 350,000 flights and can cut up to 80% of the life cycle emissions of jet fuel. However, SAF is currently three times more expensive than petroleum jet fuel and the supply has been a bottleneck restricting the wider application. A short target has been set to replace 2% and 5% of total jet fuel consumption by SAF by 2025 and 2030, respectively.

Fig. 4 in the main text first presents a projection of the cumulative well-to-wake baseline emissions for international aviation from 2019 to 2050, which are estimated to be 44 $GtCO_2e$. The following are the steps taken to obtain this estimate. The annual global jet fuel consumption data from 2005 to 2021 are obtained from the IEA World Oil Statistics[33], ranging from 233 to 333 million tonnes per year. The annual global jet fuel consumption in 2022, 2023, and 2024 are projected to be at the same level as 2015, 2018, and 2019 respectively[33]. Jet fuel use from 2025 to 2050 is assumed to follow the growth in jet fuel demand projected by the ICAO[34] scenario of

1061 million tonnes per year between 2024 and 2050. The impact of fleet renewal and increasing fleet efficiency leading to reduced jet fuel consumption is considered in this projection. These estimates are made for domestic and international aviation combined. To investigate the impacts of international aviation alone, a fixed proportion of international to total jet fuel consumption of 57% (based on IEA 2017 data[33]) is assumed for all years. That is, every year, 57% of the total global jet fuel use is assumed to be used for international aviation. By converting the well-to-wake GHG emissions of 88.7 gCO_2e MJ^{-1} jet fuel presented in this study, we can get a life cycle emissions factor of 3.8 tonne CO_2e per tonne jet fuel (88.7 gCO_2e/MJ * 0.001 kg/g * 42.8 MJ/kg jet fuel = 3.8 tonne CO_2e/tonnes jet fuel). This emissions factor is then applied to get the well-to-wake GHG emissions (the thick solid line in Fig.4) from international aviation between 2005 and 2050. By summing up the numbers for every year between 2019 and 2050 (2019 is picked as the start of implementing emissions reductions), the cumulative jet fuel consumption and well-to-tank emissions from international aviation are 11.5 Gt jet fuel and 44 $GtCO_2e$, respectively. Note there are other mitigation opportunities that should be explored in future analyses as well shifts in demand that could affect these trajectories.

The dashed line represents ICAO's aspirational goal for international aviation of carbon neutral growth from 2019 levels, which is 727 $GtCO_2e$ per year to 2050 (note that the original emissions baseline for CORSIA's carbon-neutral growth target is the average of 2019 and 2020, which was amended to the 2019 emission levels due to COVID-19)[35]. The first two reduction categories are aircraft technology improvements and improved air traffic management (ATM) and infrastructure use, which have been in effect for some years. Aircraft technology improvements are aimed at propulsion, aerodynamics, weight reduction, and urban air mobility; ATM refers to safe and orderly traffic flow to avoid congestion and efficient airspace management for both civil and military use; and infrastructure use refers to operational improvement (e.g., ensuring the plane's engines are clean to developing and using new arrivals procedures). There have been several studies estimating potential fleet wide emissions benefits of aircraft and air traffic technology improvements in the literature[36–39]. In this study, according to the ICAO's 2019 environment report, we set the fuel use reductions from aircraft technology and operation (ATM and infrastructure use) improvements as 0.98% and 0.39% per annum[34]. We then calculate the annual fuel use and GHG emissions savings (i.e., fuel use times 3.8 tonne CO_2e per tonne jet fuel as above) from both improvements by applying these exponentially decreasing rates for the period of 2019-2050, which are shown as the blue (7.8 $GtCO_2e$) and green areas (2.8 $GtCO_2e$) under the baseline curve in Fig. 4.

The potential GHG emissions savings from decarbonizing the jet fuel supply chain (see Methods, Jet fuel supply chain GHG emissions reduction technologies and scenarios, low and high technology improvements scenarios) can be 0.7 and 2.3 $GtCO_2e$ by 2050. These savings only start from 2026 because that is when the predicted annual emissions less GHG savings from technology and infrastructure improvements are above the ICAO's carbon-neutral growth target. The GHG savings from the low and high technology improvements scenarios can be calculated by comparing their well-to-wake CI (i.e., 86.6 and 81.6 gCO_2e MJ^{-1} jet fuel for low and high technology improvements, respectively) to the baseline CI of 88.7 gCO_2e MJ^{-1} jet fuel and

multiplying the CI differences with annual jet fuel use. Note that the low technology improvements scenario is a subset of the high technology improvements scenario.

The remaining emissions reductions required for the ICAO's carbon-neutral growth target (727 GtCO2e per year) would be 8.8 $GtCO_2e$ by 2050, if the high technology improvements are applied toward the jet fuel supply chain. This number would be 11.1 $GtCO_2e$ if no supply chain decarbonisation is adopted. Such remaining reductions can be achieved via methods like carbon offsetting and low-carbon aviation fuels that meet specific sustainability criteria. Therefore, to keep every year's emissions at the ICAO's carbon-neutral growth target, a cumulative emission of 21.6 $GtCO_2e$ needs to be reduced between 2019 and 2050.

References

1. Gan, Y. *et al.* Carbon footprint of global natural gas supplies to China. *Nat. Commun.* **11**, (2020).
2. IEA. Methane from oil & gas. https://www.iea.org/reports/methane-tracker-2020/methane-from-oil-gas.
3. Nie, Y. *et al.* Greenhouse-gas emissions of Canadian liquefied natural gas for use in China: Comparison and synthesis of three independent life cycle assessments. *J. Clean. Prod.* **258**, 120701 (2020).
4. Liu, R. E. Life Cycle Greenhouse Gas Emissions of Western Canadian Natural Gas and a Proposed Method for Upstream Life Cycle Emissions Tracking. (2019) doi:10.11575/PRISM/10182.
5. Lechtenböhmer, S., Dienst, C. & Lechtenbo¨hmer, S. L. Future development of the upstream greenhouse gas emissions from natural gas industry, focussing on Russian gas fields and export pipelines. *J. Integr. Environ. Sci.* **7**, 39–48 (2010).
6. Okamura, T., Furukawa, M. & Ishitani, H. Future forecast for life-cycle greenhouse gas emissions of LNG and city gas 13A. *Appl. Energy* **84**, 1136–1149 (2007).
7. Safaei, A., Freire, F. & Henggeler Antunes, C. Life-Cycle Greenhouse Gas Assessment of Nigerian Liquefied Natural Gas Addressing Uncertainty. *Environ. Sci. Technol.* **49**, 3949–3957 (2015).
8. Choi, W. & Song, H. H. Well-to-wheel greenhouse gas emissions of battery electric vehicles in countries dependent on the import of fuels through maritime transportation: A South Korean case study. *Appl. Energy* **230**, 135–147 (2018).
9. Hammond, G. P. & O' Grady, Á. The life cycle greenhouse gas implications of a UK gas supply transformation on a future low carbon electricity sector. *Energy* **118**, 937–949 (2017).
10. PRELIM: The Petroleum Refinery Life Cycle Inventory Model. https://ucalgary.ca/energy-technology-assessment/open-source-models/prelim (2021).
11. Moretti, C., Moro, A., Edwards, R., Rocco, M. V. & Colombo, E. Analysis of standard and innovative methods for allocating upstream and refinery GHG emissions to oil products. *Appl. Energy* **206**, 372–381 (2017).
12. Argonne National Laboratory. *Updates of Hydrogen Production from SMR Process in GREET® 2019*. https://greet.es.anl.gov/files/smr_h2_2019 (2019).
13. Khojasteh Salkuyeh, Y., Saville, B. A. & MacLean, H. L. Techno-economic analysis and life cycle assessment of hydrogen production from natural gas using current and emerging technologies. *Int. J. Hydrogen Energy* **42**, 18894–18909 (2017).
14. de Mello, L. F. *et al.* A technical and economical evaluation of CO2 capture from FCC units. *Energy Procedia* **1**, 117–124 (2009).
15. Shakerian, F., Kim, K. H., Szulejko, J. E. & Park, J. W. A comparative review between amines and ammonia as sorptive media for post-combustion CO2 capture. *Appl. Energy* **148**, 10–22 (2015).
16. Stec, M. *et al.* Pilot plant results for advanced CO2 capture process using amine scrubbing at the Jaworzno II Power Plant in Poland. *Fuel* **151**, 50–56 (2015).
17. Chevron. Alternative Jet Fuels: A supplement to Chevron's Aviation Fuels Technical Review. https://www.chevron.com/-/media/chevron/operations/documents/chevron-alternative-jet-fuels.pdf (2006).
18. GEVO. *SUSTAINABLE AVIATION FUEL*. https://gevo.com/wp-content/uploads/2020/05/Gevo-Whitepaper-Sustainable-Aviation-Fuel.pdf (2019).
19. National Renewable Energy Laboratory. *Review of Biojet Fuel Conversion Technologies*.

https://www.nrel.gov/docs/fy16osti/66291.pdf (2016).

20. MIT. LCA of Current & Future GHG Emissions from Petroleum Jet Fuel. https://www.energy.gov/sites/prod/files/2016/09/f33/speth_alternative_aviation_fuel_workshop.pdf (2016).

21. Zhou, W., Wang, T., Yu, Y., Chen, D. & Zhu, B. Scenario analysis of CO2 emissions from China's civil aviation industry through 2030. *Appl. Energy* **175**, 100–108 (2016).

22. ICAO. SARPs - Annex 16 Volume IV. https://www.icao.int/environmental-protection/CORSIA/Pages/SARPs-Annex-16-Volume-IV.aspx (2018).

23. Calzado Catalá, F. *et al.* Estimating the marginal CO2 intensities of EU refinery products. *CONCAWE Reports* (2017).

24. Cooney, G. *et al.* Updating the U.S. life cycle GHG petroleum baseline to 2014 with projections to 2040 using open-source engineering-based models. *Environ. Sci. Technol.* **51**, 977–987 (2017).

25. ICAO. *CORSIA Default Life Cycle Emissions Values for CORSIA Eligible Fuels*. https://www.icao.int/environmental-protection/CORSIA/Documents/ICAO document 06 - Default Life Cycle Emissions - March 2021.pdf (2021).

26. Wood Mackenzie. Oil Refining & Marketing. https://www.woodmac.com/our-expertise/capabilities/oil-refining-marketing/ (2017).

27. Wood Mackenzie. Refinery Benchmarking Tool. https://www.woodmac.com/research/products/oils-refining-ngls/refinery-evaluation-model/ (2019).

28. Elgowainy, A. *et al.* Energy efficiency and greenhouse gas emission intensity of petroleum products at U.S. Refineries. *Environ. Sci. Technol.* **48**, 7612–7624 (2014).

29. Argonne National Laboratory. *Life-Cycle Analysis of Alternative Fuels in GREET*. https://publications.anl.gov/anlpubs/2016/05/127787.pdf (2012).

30. Jang, J. J. & Song, H. H. Well-to-wheel analysis on greenhouse gas emission and energy use with petroleum-based fuels in Korea: gasoline and diesel. *Int. J. Life Cycle Assess.* **20**, 1102–1116 (2015).

31. Nimana, B., Canter, C. & Kumar, A. Life cycle assessment of greenhouse gas emissions from Canada's oil sands-derived transportation fuels. *Energy* (2015) doi:10.1016/j.energy.2015.05.078.

32. Jing, L. *et al.* Carbon intensity of global crude oil refining and mitigation potential. *Nat. Clim. Chang.* (2020) doi:10.1038/s41558-020-0775-3.

33. IEA. World Oil Statistics. https://www.iea.org/data-and-statistics/data-product/oil-information#world-oil-statistics (2020).

34. ICAO. *2019 Environmental Report*. https://www.icao.int/environmental-protection/pages/envrep2019.aspx (2019).

35. ICAO. Impact of COVID-19 on CORSIA implementation in 2020. https://www.icao.int/environmental-protection/CORSIA/Pages/CORSIA-and-Covid-19.aspx (2020).

36. Moolchandani, K., Govindaraju, P., Roy, S., Crossley, W. A. & De Laurentis, D. A. Assessing effects of aircraft and fuel technology advancement on select aviation environmental impacts. in *Journal of Aircraft* (2017). doi:10.2514/1.C033861.

37. Hassan, M., Pfaender, H. & Mavris, D. Probabilistic assessment of aviation CO2 emission targets. *Transp. Res. Part D Transp. Environ.* (2018) doi:10.1016/j.trd.2018.06.006.

38. Larsson, J., Elofsson, A., Sterner, T. & Åkerman, J. International and national climate policies for

aviation: a review. *Climate Policy* (2019) doi:10.1080/14693062.2018.1562871.

39. Van Vuuren, D. P. *et al.* Alternative pathways to the 1.5 °c target reduce the need for negative emission technologies. *Nat. Clim. Chang.* (2018) doi:10.1038/s41558-018-0119-8.
